# The improved cure fraction for esophageal cancer in Linzhou city

**DOI:** 10.1186/s12885-018-4867-7

**Published:** 2018-10-03

**Authors:** Shuzheng Liu, Lanwei Guo, Qiong Chen, Liang Yu, Bianyun Li, Xiaoqin Cao, Xibin Sun

**Affiliations:** 1 Henan Cancer Research and Control Office, Affiliated Tumor Hospital of Zhengzhou University, Henan Tumor Hospital, Zhengzhou, 450008 China; 2 Linzhou Cancer Registry, Linzhou Cancer Hospital, Linzhou, 456500 China

**Keywords:** Esophageal cancer, Survival analysis, Cure model

## Abstract

**Background:**

Survival of esophageal cancer in Linzhou was seen to increase over the past few decades and is higher than the average level of China due to the implementation of comprehensive prevention and control measures. In population-based studies, relative survival is a common index to approximate disease-specific survival. However, the cure fraction maybe great interest to patients and physicians. This study aimed to investigate the cure fraction of esophageal cancer in Linzou city during 2003–2012 with a cure model.

**Methods:**

We carried out a population-based study of 8067 esophageal cancer patients in the Linzhou city during 2003–2012. Flexible parametric cure models were used to estimate cure proportions and median survival times of uncured by year of diagnosed and age. In each model, an interaction between calendar year and age were included. All variables in the model were included both as constant and time-varying effects.

**Results:**

The 5-year relative survival rate was increased in every age group from 2003 to 2012. The huge increase in the cure proportion was observed in each age group. At the year of 2011–2012, 79.8%, 58.0%, 123.4% and 162.7% improvements of cure proportion were seen in age group 19–49, 50–59, 60–69 and 70–99 years compared with year of 2003–2004. Meanwhile, survival of ‘uncured’ patients changed little in all age group.

**Conclusions:**

The improvement of survival in Linzhou city during 2003–2012 was mainly due to an increasing cure proportion. Huge improvement of cure fraction within short period is likely due to the organized screening of esophageal cancer in Linzhou city.

## Background

Esophageal cancer is the eighth most common cancer around the world and the fifth most common cancer in China [1, 2]. There is a large geographical variation with an incidence rate roughly 3 times higher in the incidence rates in low urbanization areas than high urbanization areas in China [3]. Linzhou city has high incidence of esophageal cancer in China. An organized screening of esophageal cancer in population was carried out since 2005. Survival of esophageal cancer in Linzhou was seen to increase over the past few decades and was higher than the average level of China [4–6]. Population-based studies of esophageal cancer survival are meaningful for clinicians, patients and health administrators.

In the population-based cancer registries, the survival for patients is traditionally measured using the cumulative survival function (cause-specific survival or relative survival) [7, 8]. However, for patients and physicians, the cure fraction may be of great interest. Cure models can be used to calculate both the cure proportion and the survival function of “uncured” patients [9]. When looking at temporal changes, more detailed view of the changes observed in the survival of cancer patients can be obtained with cure model than traditional methods.

We study the survival of patients of esophageal cancer in Linzhou city from 2003 to 2012, using cure model.

## Methods

### Data

Data were extracted from the database of Linzhou Cancer Registry. The data of incidence of esophageal cancer was routinely linked to the database of vital statistic system to obtain date and cause of death information. Those unmatched cancer patients will be followed up annually. We studied patients diagnosed as esophageal cancer in the period 2003–2012. And patients were excluded if the cancer incidence was only recorded on the information on death certificates. All patients were followed up until death, emigration or 31th December, 2016.

### Statistical methods

The relative survival is used commonly in the studies of population-based cancer registry as the measure of cancer patient survival. Relative survival is calculated as the observed survival divided by the expected survival in the disease-free population. When using relative survival the information of death cause is not needed and relative survival captures mortality directly and indirectly.

In cure model the patients diagnosed with cancer can be divided into two groups: cured patients (statistical cure) and uncured patients. The cured patients have the same mortality rate with the people of the same age and sex in the general population. So the cured patients will not experience excess mortality due to the cancer. And the cure proportion is defined as the proportion of cured patients in all patients. The point of statistical cure happens when the curve of cumulative relative survival shows a plateau and the proportion at the point is defined as the estimated cure fraction. The concept of statistical cure is different from medical cure at an individual level and used at a grouped level. Cure models can be used to calculate the cure proportion of the cured patients and the survival function of the uncured patients.

### Modelling approach

The flexible parametric cure model, a special case of a non-mixture cure model, was fitted in this study [10–12]. And instead of using a specific parametric distribution, the shape of the survival distribution was modeled with restricted cubic spline [13]. The flexible parametric cure model has been seen to fit better than the mixture and non-mixture model [9, 10, 14]. For cure fraction and median survival of the uncured group, calendar years were modeled continuously using splines [15] or 5 calendar periods (2003–2004, 2005–2006, 2007–2008, 2009–2010, 2011–2012) and age was divided in four categories (19–49, 50–59, 60–69 and 70–99). In each model, an interaction between calendar year and age were included. And all variables in the model were included both as constant and time-varying effects. To assess the fit of the cure models, we compared the estimated relative survival from the flexible parametric cure model with empirical life table estimates of relative survival using the Ederer II method [16]. All analysis was performed with the command of stpm2 [17] in stata12.0.

## Results

There were 8067 patients (56% males and 44% females) with esophageal cancer in this study. The average age at diagnosis was 62 years. The relative survival rate was 36.3%, 33.5%, 26.8% and 17.8% in age group 19–49, 50–59, 60–69 and 70–99 years respectively during 2003–2004. At the year of 2011–2012 the rate was severally 62.5%, 49.0%, 51.1 and 41.7% and there was 72.2%, 46.3%, 90.7% and 34.3% improvements respectively compared with year of 2003–2004. Demographic characteristics and survival indicator were shown in Table 1.

**Table 1:** Demographic features of patients diagnosed with esophageal cancer in Linzhou city 2003–2012

Calendar period	2003–2004	2005–2006	2007–2008	2009–2010	2011–2012	Total
Total No. of cases (%)	1439 (17.8)	1435 (17.8)	1730 (21.4)	1740 (21.6)	1723 (21.4)	8067 (100.0)
Sex, n (%)						
Male	814 (17.9)	783 (17.2)	1013 (22.3)	943 (20.7)	995 (21.9)	4548 (100.0)
Female	625 (17.8)	652 (18.5)	717 (20.4)	797 (22.6)	728 (20.7)	3519 (100.0)
Age groups, years; n (%)						
19–49	182 (24.0)	160 (21.1)	155 (20.5)	120 (15.9)	140 (18.5)	757 (100.0)
50–59	465 (18.7)	449 (18.0)	582 (23.4)	534 (21.4)	460 (18.5)	2490 (100.0)
60–69	408 (15.7)	431 (16.6)	533 (20.5)	593 (22.8)	634 (24.4)	2599 (100.0)
70–99	384 (17.3)	395 (17.8)	460 (20.7)	493 (22.2)	489 (22.0)	2221 (100.0)
Median age at diagnosis	62	61	61	62	63	62
5 year Relative survival rate (%)						
19–49	36.3	38.7	50.9	51.4	62.5	46.5
50–59	33.5	39.1	43.9	41.4	49.0	41.6
60–69	26.8	28.7	32.7	42.9	51.1	37.7
70–99	17.8	22.0	25.7	31.7	41.7	28.3

**Fig. 1:**
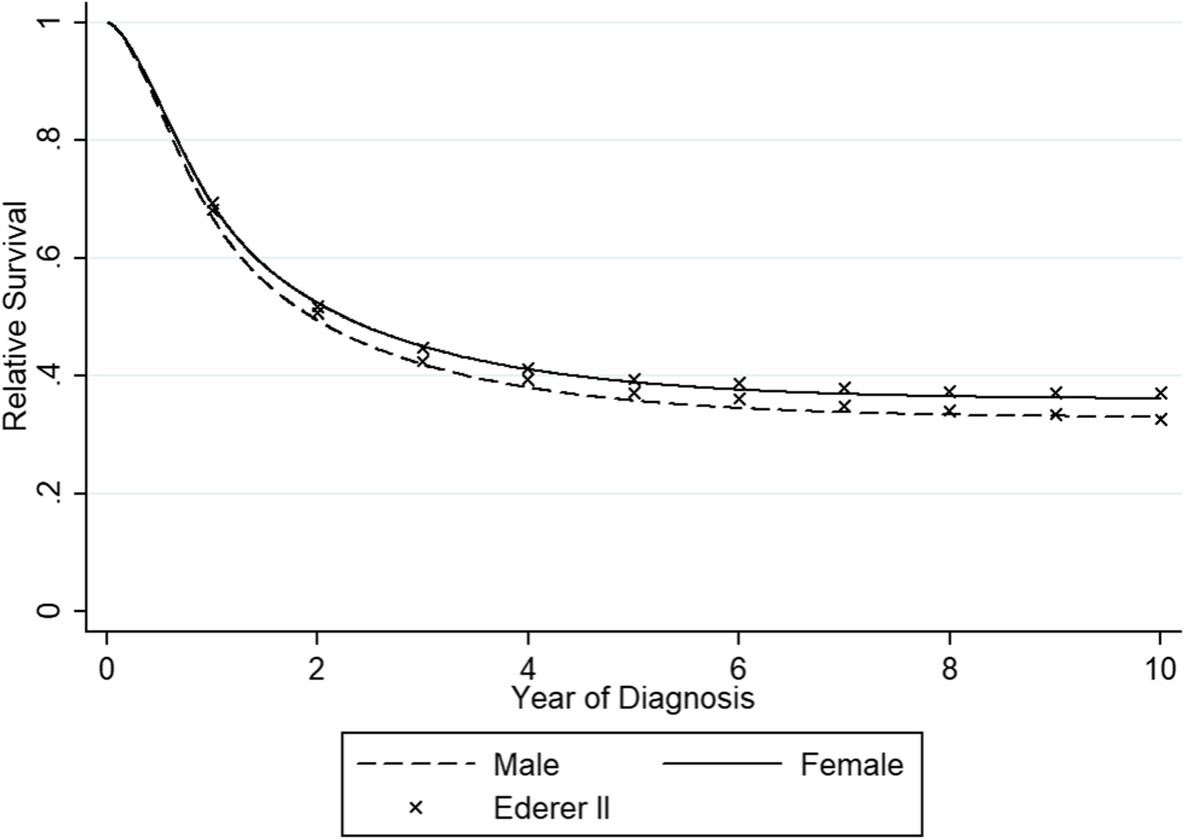
Predicted relative survival by sex

First, models were fitted by sex separately. The fitted curves of the estimated relative survival rate for male and female can be seen in Fig. 1. The empirical life table estimates calculated with the Ederer II method are also shown in the figure. It can be seen that the flexible parametric cure model fits the data well. After 5 years of follow-up the curves of relative survival rate tend to reach a plateau.

**Fig. 2:**
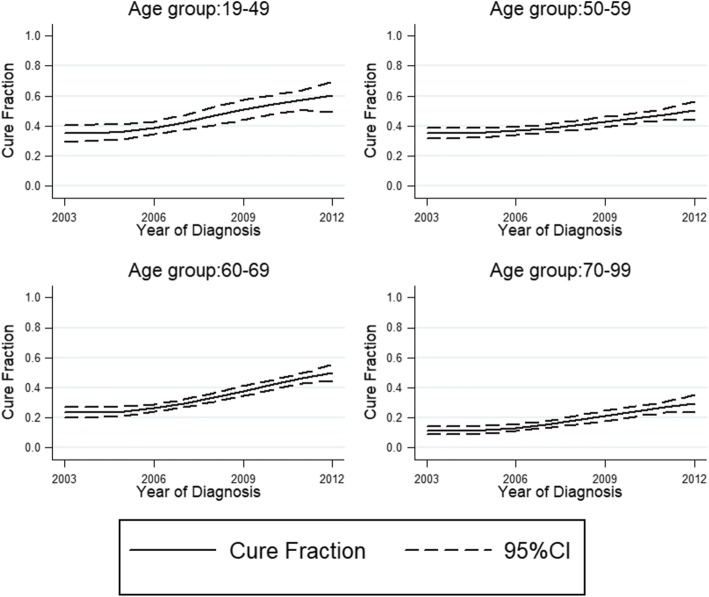
Estimated cure fraction with 95% confidence intervals presented by age group

During 2003–2004 period, the cure proportion was 33.7%, 31.4%, 21.8% and 11.0% in age group 19–49, 50–59, 60–69 and 70–99 (Table 2, Fig. 2) and at the year of 2011–2012 the proportion was 60.6%, 49.6%, 48.7% and 28.9% separately. There was constant improvement of cure proportion with time going on in all age groups. At the year of 2011–2012, 79.8%, 58.0%, 123.4% and 162.7% improvements of cure proportion were seen in age group 19–49, 50–59, 60–69 and 70–99 years compared with year of 2003–2004.

**Table 2:** Cure proportion(%), median survival time of ‘uncured’ (years) and time at which 90% of ‘uncured’ are dead (year), 95% confidence intervals, for patients diagnosed with esophageal cancer in Linzhou city 2003–2012

Year	Age group(years)			
	19–49	50–59	60–69	70–99
Cure proportion (%)				
2003–2004	33.7 (27.0–40.5)	31.4 (27.2–35.7)	21.8 (17.9–26.0)	11.0 (8.0–14.4)
2005–2006	34.4 (27.4–41.6)	38.1 (33.5–42.7)	25.7 (21.5–30.0)	14.6 (11.0–18.5)
2007–2008	49.6 (41.5–57.2)	42.2 (38.0–46.3)	31.8 (27.7–36.0)	18.1 (14.4–22.1)
2009–2010	49.7 (40.5–58.3)	41.3 (37.1–45.6)	40.4 (36.1–44.6)	19.7 (16.0–23.7)
2011–2012	60.6 (51.6–68.4)	49.6 (44.7–54.4)	48.7 (44.3–52.9)	28.9 (24.3–33.6)
Median survival of ‘uncured’ (years)				
2003–2004	1.14 (1.01–1.27)	1.19 (1.10–1.27)	1.02 (0.94–1.10)	0.75 (0.68–0.81)
2005–2006	1.13 (1.00–1.27)	1.23 (1.14–1.33)	1.05 (0.97–1.14)	0.79 (0.72–0.86)
2007–2008	1.23 (1.08–1.37)	1.24 (1.15–1.33)	1.09 (1.00–1.17)	0.81 (0.74–0.88)
2009–2010	1.28 (1.13–1.43)	1.28 (1.19–1.38)	1.21 (1.12–1.30)	0.86 (0.79–0.93)
2011–2012	1.15 (0.99–1.31)	1.16 (1.06–1.26)	1.08 (0.98–1.17)	0.78 (0.70–0.85)
Time at which 90% of the ‘uncured’ are dead (years)				
2003–2004	3.67 (3.41–3.93)	3.70 (3.50–3.89)	3.30 (3.09–3.51)	2.52 (2.29–2.74)
2005–2006	3.67 (3.40–3.94)	3.85 (3.65–4.05)	3.42 (3.21–3.63)	2.71 (2.49–2.95)
2007–2008	3.95 (3.69–4.21)	3.90 (3.71–4.10)	3.57 (3.38–3.77)	2.85 (2.63–3.08)
2009–2010	4.02 (3.76–4.28)	3.95 (3.76–4.14)	3.85 (3.65–4.04)	3.00 (2.78–3.22)
2011–2012	3.93 (3.65–4.21)	3.86 (3.64–4.08)	3.75 (3.52–3.97)	3.02 (2.77–3.27)

**Fig. 3:**
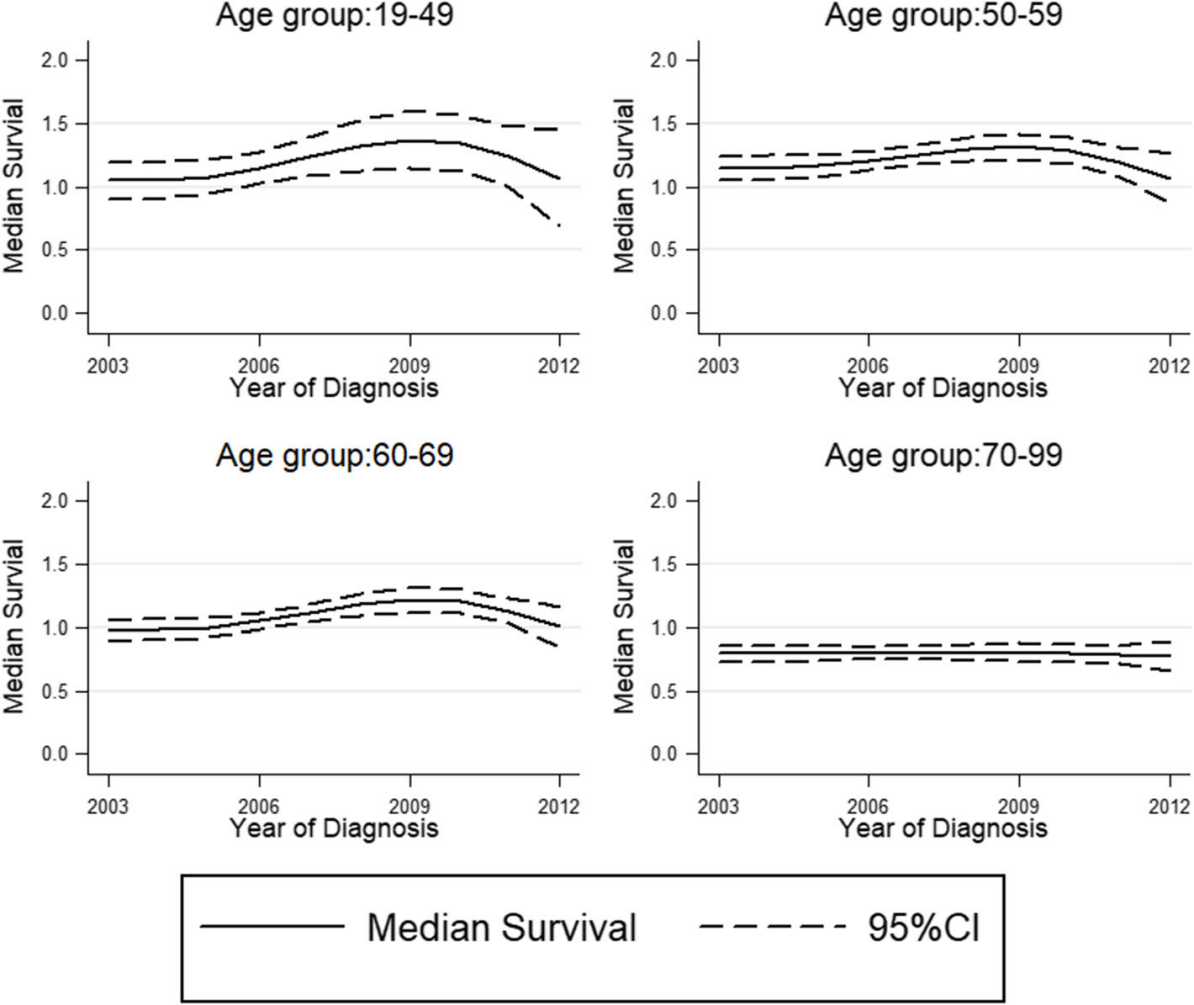
Median survival of the uncured group with 95% confidence intervals presented by age group

At the same time, median survival of ‘uncured’ patients changed little during different period. There were separately 0.01, − 0.03, 0.06 and 0.03 years difference in median survival time for age group 19–49, 50–59, 60–69 and 70–99 from 2003 to 2012. The time at which 90% of the ‘uncured’ patients are dead, which is another indicator for measuring survival of the ‘uncured’ has little variation in every age group (Table 2, Fig. 3).

## Discussion

The 5 year relative survival rate was increased in each age group from 2003 to 2012. The cure fraction has increased greatly over time for esophageal cancer in Linzhou city during 2003–2012 with very little variation of median survival of uncured group at the same time. The improvement in survival was mainly attribute to an increasing proportion of ‘cured’ patients.

When studying trends with cure models considering the cure fraction and the survival time of the uncured patients at the same time, better answer will be probably distinguished from many competing explanations [18]. Higher cure proportion and longer survival time of uncured represents a general improvement. Higher cure proportion and shorter survival time of uncured represents a selective improvement: improvements in treatment enabled uncured patients with a longer survival time to be cured. Stable cure proportion and longer survival time of uncured might occur due to better palliative care or new diagnostic techniques introduced which can move up the diagnosed date but without affecting the date of death, that is, lead time bias. Higher cure proportion and unchanged survival time of uncured might occur following introduction of some diagnostic procedure, like screening, which included more patients who have no excess risk.

An organized screening of esophageal cancer was carried out for people aged 40–69 since 2005 in Linzhou city. From 2006 to 2012, 22,869 people took part in the screening of esophageal cancer [19]. There were higher proportion of early-stage esophageal cancer cases and 5 year survival rate of esophageal cancer in endoscopy screening group in Linzhou city [20, 21]. According to the screening procedure, local people aged 40–69 will be organized to screen with endoscope for esophageal cancer. The detected high-grade dysplasia and cancer of esophagus in the screening will be treated and the detected mid-grade dysplasia of esophagus will be checked 1 year later. So there should be some high-grade dysplasia or cancer of esophagus diagnosed by screening procedure in age group over 70 years. Hence the survival of esophageal cancer aged over 70 years can also be affected by the screening. An advantage of cure model is that the lead time bias has no effect on the cure fraction, so we can distinguish the true clinical benefit and lead time bias [18]. In this study, greatly improved cure fraction within short period showed a screening effect and a success in the screening of esophageal cancer in Linzhou city.

Although cure models have given us a better understanding of the survival of patients, we should keep in mind that there are some potential limitations. First, the model will still give the estimates of the cure fraction even when statistics cure is not reached. However, for esophageal cancer it seems not a problem. The cumulative relative survival for esophageal cancer was plateau after about 4 years from the date of diagnosis [4], indicating that the living patients at that time can be considered statistically cured. We have followed-up information at least 4 years which should be enough for reach a point of cure. Second, the traditional cure model may produce biased estimates in older age group due to less flexible to get the shape of the survival distribution [22]. However, the flexible parametric cure model has been shown to perform good even when older groups was included in the model [10]. In this paper, further study of cure fraction in different TNM stage cannot be done for the absence of stage variable in the data provided by Linzhou cancer registry which only collected the essential information of cancer registry during year 2003–2012.

## Conclusions

Cure model analysis of cancer survival in China has not been reported ever. In this study, the flexible parametric cure model was used to analyze the survival of esophageal cancer in Linzhou city. And we found the improvement of survival in Linzhou city during 2003–2012 was mainly due to an increasing cure proportion. Huge improvement of cure fraction within short period is likely due to the organized screening of esophageal cancer in Linzhou city.
